# AI-driven ANN–PSO smart-inverter capacity allocation at preselected buses under meteorological uncertainty

**DOI:** 10.3389/frai.2026.1896551

**Published:** 2026-08-20

**Authors:** Selman Djeffal, Badreddine Bendriss, Abdelhamid Ghoul, Mohamed Razi Morakchi

**Affiliations:** 1 Department of Mechanical Engineering, National Polytechnic School of Constantine, Constantine, Algeria; 2 QUERE Laboratory, Department of Electrical Engineering, Ferhat Abbas University, Setif, Algeria; 3 Automatic Control, Department of Computer Science, Electrical and Space Engineering, Luleå University of Technology, Luleå, Sweden; 4 Department of Electromechanics, Faculty of Construction Engineering, University of Tizi ouzzou UMMTO, Tizi Ouzou, Algeria

**Keywords:** artificial neural network, particle swarm optimization, radial distribution grid, renewable capacity allocation, scenario-based uncertainty, smart inverter, voltage stability index, Volt–VAR control

## Abstract

**Introduction:**

Radial distribution grids require renewable planning methods that jointly account for meteorological generation, WT/PV capacity allocation, and smart-inverter voltage support.

**Methods:**

An ANN-PSO-VoltVAR framework was developed using 2012-2021 NASA POWER irradiance, wind-speed, and temperature data for In Salah, Algeria. Renewable uncertainty was represented by 12 stratified Weibull-Beta scenarios and an expected-cost/CVaR objective. The mixed decision vector optimized WT units, PV strings, and voltage-dependent inverter support at four preselected IEEE 85-bus locations. An exact backward/forward-sweep module on the standard 33-bus feeder independently verified the load-flow implementation.

**Results:**

The tuned ANN achieved an RMSE of 1.6323 kW, an MAE of 1.2314 kW, and an R-squared value of 0.99999. The verified five-start mean-profile solution reduced mean active loss to 15.9900 kW and voltage deviation to 0.051082 p.u., while increasing minimum voltage to 0.98213 p.u. and minimum VSI to 0.96307. Relative to IFLO, the respective improvements were 54.57%, 22.49%, 1.21%, and 8.41%.

**Discussion:**

The ablation analysis showed that capacity sizing accounted for most of the loss reduction, whereas Volt-VAR support produced the clearer additional voltage-security gains. Because the IEEE 85-bus electrical response is benchmark-calibrated rather than fully reconstructed, the findings should be interpreted as a reproducible proof-of-concept. Direct full-feeder validation on the IEEE 85-bus system and other networks remains necessary.

## 1 Introduction

Renewable distributed generation has moved from being an auxiliary environmental option to being a decisive planning variable in modern distribution grids. The reason is electrical before it is ecological: Photovoltaic (PV) systems and wind turbines (WTs) can reduce active losses, raise weak-bus voltages, and improve the voltage stability index (VSI), but only when their capacity, location, and control mode are selected with respect to feeder constraints. Akbar et al. (2022) developed a hybrid optimization-based allocation framework and showed that distributed generation placement is not a cosmetic capacity-addition problem, but a direct determinant of network losses and voltage quality. Malik et al. (2020) proposed an advanced multi-objective PSO strategy and demonstrated that renewable planning must handle conflicting objectives because a solution that is good for loss can still be electrically weak if voltage quality is not protected. Ramadan et al. (2023) used an artificial hummingbird algorithm under uncertainty conditions and confirmed that renewable variability must be treated inside the planning model rather than corrected after the optimization. Dalawir et al. (2024) investigated wind-based distributed generators with STATCOM support and showed that renewable active-power injection becomes more valuable when it is coordinated with voltage-support capability.

The vulnerability of radial distribution grids makes this problem more severe. Al-Ammar et al. (2021) used an artificial bee colony algorithm for distributed generation sizing and placement, showing that the loss-reduction surface is highly non-linear and sensitive to candidate location. Tolba et al. (2020) reviewed heuristic techniques for connecting renewable distributed generators and emphasized that voltage limits, losses, and stability margins must be handled together. Venkatesan et al. (2021) proposed a multi-objective hybrid method for distributed generation and compensation devices, underlining that reactive support can alter the technical quality of the final plan. Ba-swaimi et al. (2024) studied long-term planning of distributed generation and storage under uncertainty and demand response, showing that renewable planning becomes more realistic only when uncertainty and operating conditions are explicitly represented. These studies collectively make one point unavoidable: A radial feeder can receive renewable power and still remain poorly planned if the optimizer does not see weather variability, network limits, and voltage-support functions at the same time.

The direct benchmark of the present study is the improved Frilled Lizard Optimization (IFLO) formulation proposed by Bendriss et al. (2024) for WT/PV planning in the IEEE 85-bus radial distribution system. That benchmark is important because it used seasonal meteorological profiles, Weibull wind modeling, Beta solar modeling, and time-dependent loading, and because it reported clear electrical gains. In that reference case, the mean active loss decreased from 118.2953 kW to 35.1939 kW, the voltage deviation decreased from 0.30662 p.u. to 0.06590 p.u., the minimum voltage increased from 0.9252 p.u. to 0.9704 p.u., and the minimum VSI improved from 0.7355 to 0.8884. These values define a serious baseline, but they also expose the central limitation that motivates this study: The benchmark remains mainly a probabilistic sizing framework, while modern renewable plants are data-driven and power-electronic resources. It does not directly learn the non-linear weather-to-power mapping from real hourly data, and it does not optimize PV inverter reactive-power support as an active planning variable.

Several optimizer-centered studies have improved renewable allocation, but most of them still leave this forecasting–control coupling underexploited. Reddy et al. (2017) used the Whale Optimization Algorithm for renewable sizing and loss reduction, confirming the ability of population-based algorithms to search difficult distribution-planning spaces. Mirjalili and Lewis (2016) introduced the Whale Optimization Algorithm itself as a general metaheuristic, providing the algorithmic basis for later power-system applications. Gumus et al. (2023) used a stability-index-driven allocation approach and showed that voltage stability should be treated as a central planning objective. Pamuk et al. (2024) applied the Arithmetic Optimization Algorithm to distributed generation and compensation allocation, again showing that the planning decision is mixed, non-linear, and constrained. Khasanov et al. (2021) considered distributed generation and battery storage under generation uncertainty, reinforcing the need to account for renewable variability. Jagtap et al. (2024) used a pseudo-inspired gravitational search algorithm for distribution planning under uncertainty, confirming that robust performance requires more than deterministic sizing. Avar et al. (2024) combined analytical techniques, metaheuristics, and deep learning for PV/wind integration, showing that learning-assisted renewable planning is becoming technically necessary rather than optional. Bendriss et al. (2023) proposed an adaptive PSO variant for distributed generation optimization, supporting the continued relevance of PSO-family algorithms when the decision space mixes discrete and continuous variables.

Recent hybrid-renewable EV-charging studies also clarify the boundary of the present contribution. Soomro et al. (2024) performed a techno-economic analysis of a stand-alone hybrid PV–hydrogen plug-in electric-vehicle charging station. Their study is valuable because it demonstrates how PV and hydrogen storage can be sized from an energy supply and economic perspective. However, the problem is formulated at the charging-station system level, not as a radial distribution-feeder planning problem with bus-voltage constraints and inverter reactive-power decision variables.

Similarly, Kumar et al. (2024) compared off-grid PV–wind–hydrogen electric-vehicle charging stations under four climatically distinct Pakistani cities. This study is relevant because it shows that renewable-resource complementarity and climate-dependent sizing strongly influence the optimal energy-system configuration. The present study uses this insight but moves the question from off-grid station design to grid-aware WT/PV capacity allocation at preselected buses, where the final decision must also reduce feeder loss, voltage deviation, and voltage-stability risk.

At the distribution-network level, Memon et al. (2023) proposed a deterministic methodology to enhance radial distribution-network performance while increasing plug-in electric-vehicle accommodation and reducing power loss. This study is important because it links transportation electrification with distribution-network technical limits. Nevertheless, it does not address the weather-driven WT/PV generation-estimation layer or the simultaneous optimization of renewable-source sizing and PV-inverter Volt–VAR support.

For metaheuristic DG allocation, Memon et al. (2022) developed a parallel hybrid Arithmetic–Salp swarm optimizer for allocating multiple distributed generation units in distribution networks. That study confirms the value of hybrid swarm search for DG siting and sizing. The present study differs by not claiming novelty from a new swarm variant; instead, it uses PSO as the search engine inside a coupled ANN–PSO–VoltVAR architecture in which active-power capacity allocation and reactive-power inverter support are optimized together at preselected buses.

Other works focused more directly on voltage security and uncertainty handling. Barnwal et al. (2022) developed a multi-objective approach for voltage-stability enhancement and loss reduction through reconfiguration and DG allocation, showing that stability reinforcement must be part of the objective rather than a secondary output. Selim et al. (2020) studied voltage-stability-based DG placement and showed that the final plan can change significantly when stability indices are considered. Selim et al. (2024) proposed a modified Runge–Kutta optimization for PV and battery allocation under uncertainty and load variation, highlighting the importance of time-varying operating conditions. Jamal et al. (2023) addressed optimal reactive power dispatch with renewable sources, demonstrating that reactive-power decisions strongly influence renewable-rich operation. Ahmed et al. (2020) proposed a probabilistic wind generation model for optimal allocation, confirming that stochastic renewable availability affects optimal placement. Abdel-Mawgoud et al. (2021) considered PV and battery allocation under uncertainty using a modified optimizer, showing that storage and uncertainty models can improve planning realism. These works are valuable, but they do not close the exact gap attacked here: a single planning chain that learns real meteorological generation, optimizes WT/PV capacity at preselected buses, and activates PV inverter Volt–VAR support to beat a strong benchmark simultaneously in loss, voltage deviation, minimum voltage, and VSI.

The artificial-intelligence layer is the decisive difference in this study. Hornik et al. (1989) established the universal approximation capability of multilayer neural networks, which supports the use of ANN models for non-linear weather-to-generation mapping. Breiman (2001) introduced Random Forests and showed the value of ensemble learning for non-linear tabular prediction. Rasmussen and Williams (2006) presented Gaussian processes as a principled regression framework, making them a useful smooth non-linear baseline. Kennedy and Eberhart (1995) introduced particle swarm optimization, whose simple velocity–position update remains attractive for mixed planning variables. In the present study, these ideas are not used as isolated tools. The ANN estimates renewable availability from weather variables, the PSO searches mixed WT/PV and inverter-support variables, and the Volt–VAR layer transforms the PV inverter from a passive generator into a voltage-support actuator.

The research gap addressed in this study is therefore specific. Previous renewable-planning studies have usually emphasized one of three elements: probabilistic resource modeling, metaheuristic source allocation, or voltage/reactive-power support. What remains less explicit is a single planning chain in which the renewable-generation estimator, the mixed-integer capacity-allocation variables, and the smart-inverter reactive-power variables are optimized together and judged by simultaneous loss, voltage-deviation, minimum-voltage, and VSI criteria. The proposed method is not presented as a new Weibull model, a new Beta model, a new ANN, or a new PSO variant. Its contribution lies in the closed integration of these established components into a planning architecture that makes inverter support part of the planning decision rather than a corrective action added after source sizing.

This distinction also clarifies the difference from earlier ANN–PSO planning schemes. In many ANN–PSO studies, the ANN is used only as a predictor or the PSO is used only for active-power sizing. Such a structure cannot demonstrate whether the final gain comes from better forecasting, from metaheuristic tuning or from an additional voltage-control actuator. Here, the decision vector explicitly contains WT units, PV strings, and PV inverter Volt–VAR ratios; consequently, the optimization is mixed discrete–continuous and physically connected to the voltage profile. The strict research question is: how much of the reported electrical improvement is attributable to capacity allocation, the ANN surrogate and voltage-dependent inverter support, and can these contributions be separated transparently?

The main contributions are therefore as follows. First, a real-data-assisted renewable-generation database is built from hourly NASA POWER irradiance, wind speed, and temperature data for In Salah, Algeria (NASA Langley Research Center, 2026). Second, fitted Weibull and Beta models are propagated into 12 stratified meteorological scenarios and an expected-cost/CVaR objective. Third, the ANN total is converted into explicit bus-level WT/PV injections through candidate-dependent physical shares. Fourth, a constrained ANN–PSO–VoltVAR optimizer is formulated with four integer capacity variables at preselected buses and two continuous inverter-support limits governed by a voltage-dependent capability curve. Fifth, a controlled mean-profile ablation separates source capacity, direct physical-equation evaluation, and inverter support, while a twenty-run protocol and a capacity-matched controlled mode are supplied for reproducibility. Because the original IFLO implementation is unavailable, this controlled mode is not presented as a full algorithm-to-algorithm fairness test. The contribution is therefore integration and transparent testing, not a claim of network-wide siting or complete IEEE 85-bus validation.

## 2 Materials and methods

This section describes the complete computational chain in the same order used by the MATLAB implementation: meteorological data construction, uncertainty characterization, physical WT/PV conversion, ANN surrogate training, and mixed-variable PSO–VoltVAR planning. This linking paragraph is included to avoid an abrupt transition from the section heading to the first technical subsection.

### 2.1 Study area and data construction

**Figure 1:**
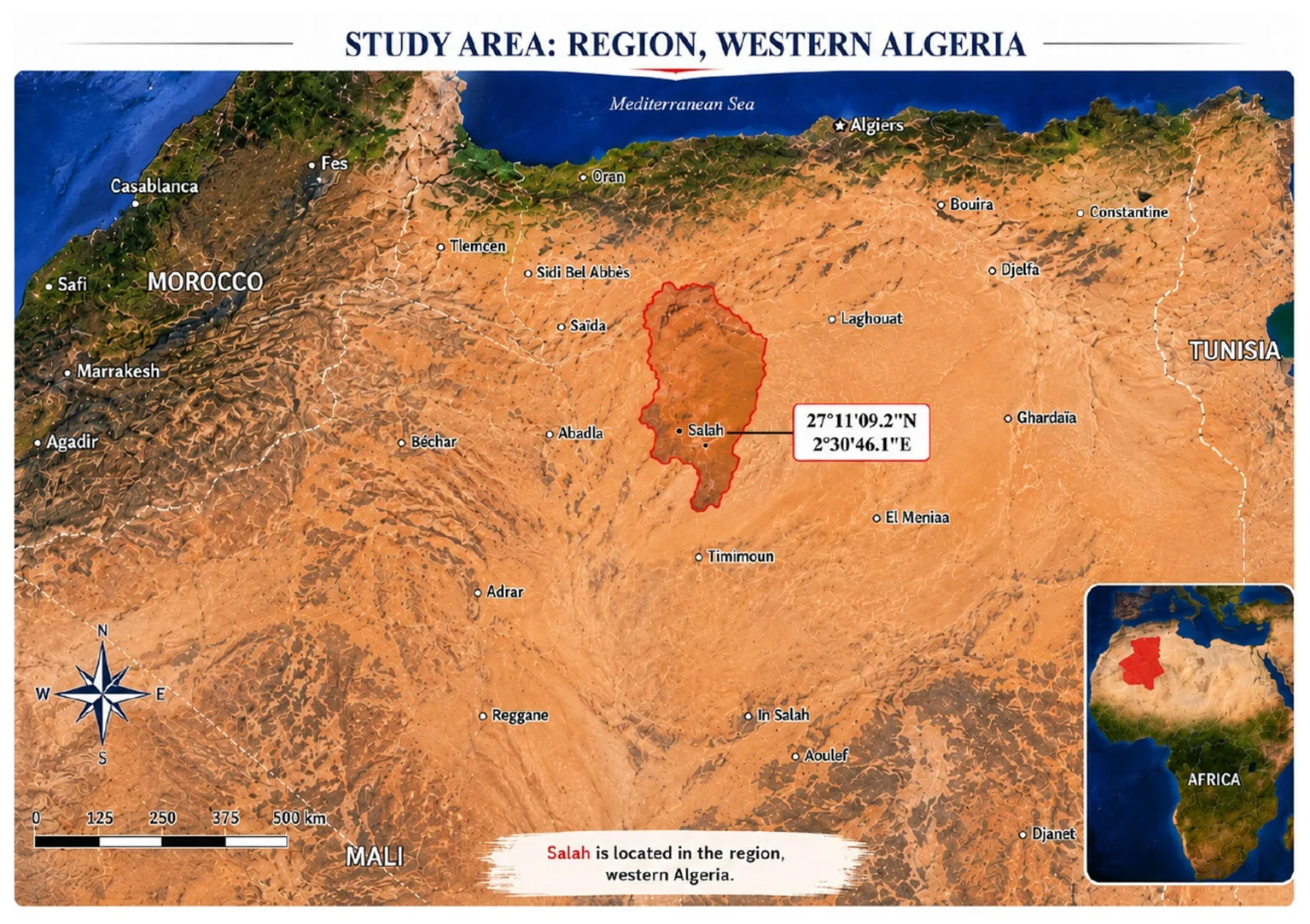
Geographical context of the Algerian study area and the In Salah meteorological point used for NASA POWER data extraction.

**Figure 2:**
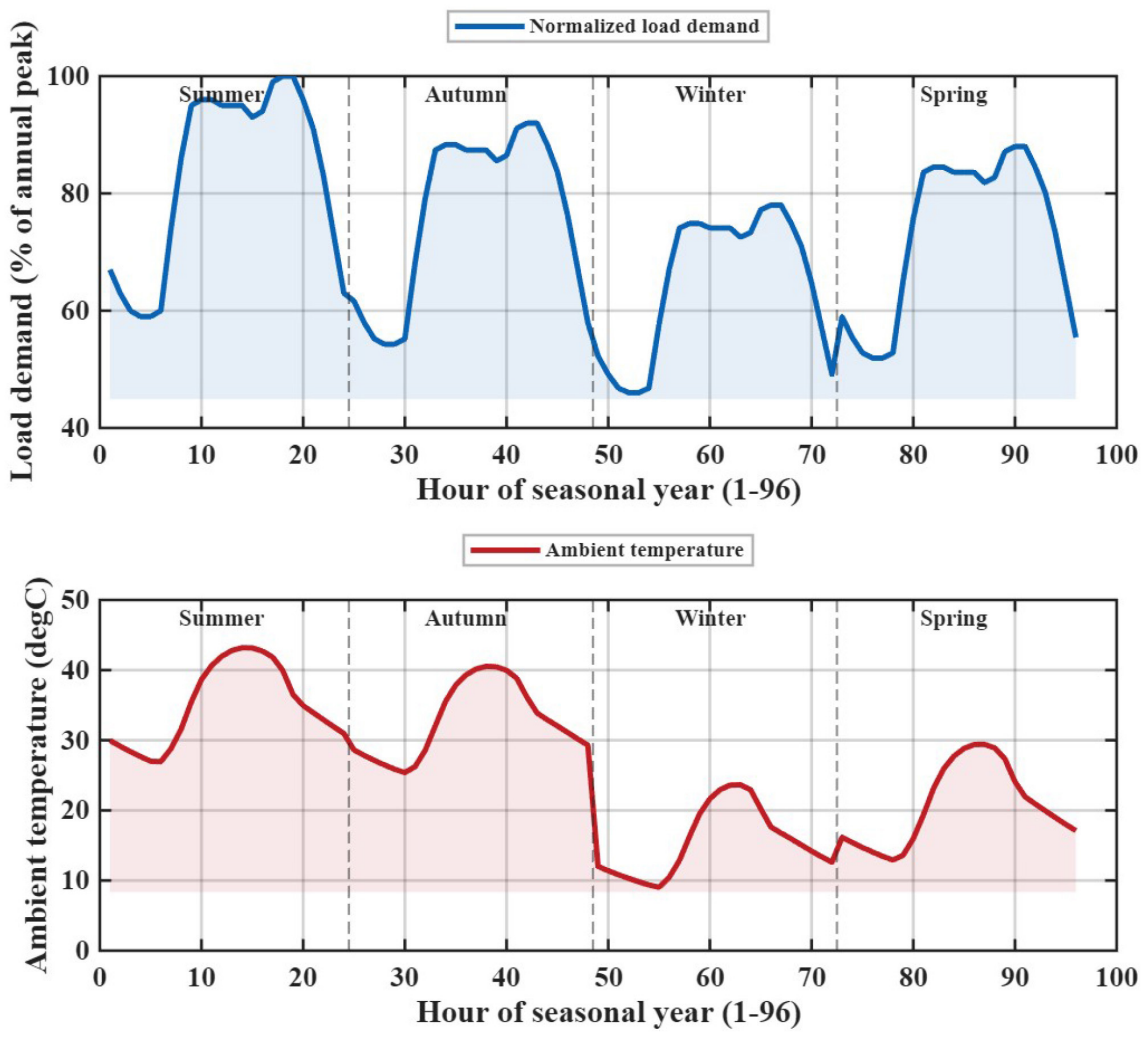
Seasonal 96-h operating inputs: normalized load demand and ambient temperature. The two panels share the same seasonal time axis used throughout the study.

The study focuses on In Salah, Algeria, a Saharan location with high renewable-energy potential. The hourly weather data cover 1 January 2012 to 31 December 2021 and include all-sky surface shortwave irradiance *G*(*t*), wind speed at 10 m *v*(*t*), and ambient temperature *T*_*a*_(*t*). The geographic context and the extracted renewable-resource profiles are summarized in Figures 1 and 2. The display-item plan was intentionally compressed: Related plots are grouped vertically so that the manuscript contains exactly 13 figures and two editable tables while keeping the evidence visible and reviewable.

Seasonal operation is represented by a 96-h profile obtained by concatenating four representative 24-h periods. Let *P*_*L*, 24_(*h*) be the base daily load percentage and α_*s*_ the seasonal multiplier for season *s*. The normalized load is


$$P_{L}(h,s)=100\frac{\alpha_{s}P_{L,24}(h)}{\max_{h,s}\left(\alpha_{s}P_{L,24}(h)\right)},h=1,\ldots ,24,s=1,\ldots ,4.$$ (1)

Equation 1 defines the common normalized load axis subsequently used for the weather, generation, loss, voltage, and VSI profiles.

### 2.2 Uncertainty and renewable-generation modeling

Wind uncertainty is represented through a Weibull probability density function:


$$\begin{array}{l} f_{v}\left(v;k,c\right)=\frac{k}{c}{\left(\frac{v}{c}\right)}^{k-1}\exp\left[-{\left(\frac{v}{c}\right)}^{k}\right],v\geq 0, \end{array}$$ (2)

where *k* and *c* are the shape and scale parameters. Solar irradiance uncertainty is represented using a Beta density:


$$\begin{array}{l} f_{g}\left(g;\alpha ,\beta \right)=\frac{\Gamma \left(\alpha +\beta \right)}{\Gamma \left(\alpha \right)\Gamma \left(\beta \right)}g^{\alpha -1}{\left(1-g\right)}^{\beta -1},0\leq g\leq 1. \end{array}$$ (3)

**Figure 3:**
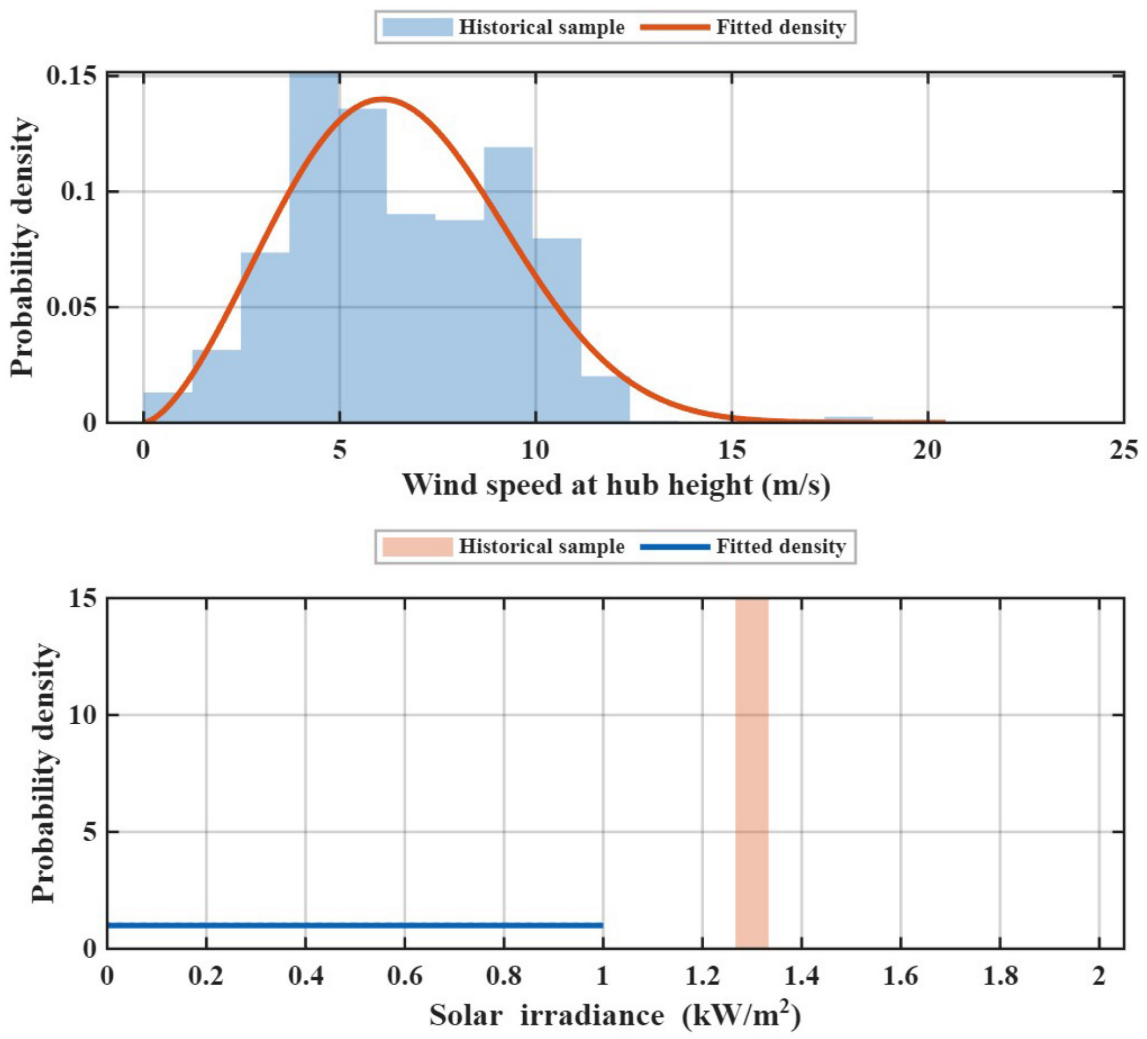
Uncertainty modeling of renewable resources: Weibull fit for wind speed and Beta fit for solar irradiance at representative seasonal hours.

The fitted distributions are shown in Figure 3. These distributions indicate that the planning problem is driven by non-uniform and season-dependent renewable availability.

The fitted distributions are also propagated into the optimization. Twelve stratified scenarios are generated with a fixed scenario seed and common random numbers. The inverse Weibull transformation is used for wind, whereas Beta quantiles are used for irradiance. For scenario *s*, the weather-dependent unit profiles are denoted as $p_{WT,s}^{u}\left(t\right)$ and $p_{PV,s}^{s}\left(t\right)$. Every candidate and every independent PSO run is evaluated on the same scenario set. The robust objective is


$$F_{rob}(X)=0.75E_{s}\left[F_{s}(X)\right]+0.25CVaR_{0.90}\left(F_{s}(X)\right)$$ (4)

where scenario-wise voltage, inverter, and curtailment violations remain penalized. Consequently, “under uncertainty” refers specifically to meteorological scenario variation within the calibrated electrical model, not to a complete probabilistic feeder model.

WT output is computed using the piecewise turbine power curve


$$\begin{array}{l} P_{WT}\left(v\right)=\{\begin{array}{ll} 0, & v<v_{ci},\\ P_{r}\frac{v^{3}-v_{ci}^{3}}{v_{r}^{3}-v_{ci}^{3}}, & v_{ci}\leq v<v_{r},\\ P_{r}, & v_{r}\leq v\leq v_{co},\\ 0, & v>v_{co}, \end{array} \end{array}$$ (5)

where *v*_*ci*_, *v*_*r*_, and *v*_*co*_ are the cut-in, rated, and cut-out wind speeds, and *P*_*r*_ is the rated turbine power. PV cell temperature and PV power are modeled as


$$\begin{array}{l} T_{c}\left(t\right)=T_{a}\left(t\right)+\frac{NOCT-20}{800}G\left(t\right), \end{array}$$ (6)

$$\begin{array}{l} P_{PV}\left(t\right)=P_{STC}\frac{G\left(t\right)}{G_{STC}}\left[1+\gamma \left(T_{c}\left(t\right)-25\right)\right]. \end{array}$$ (7)

**Figure 4:**
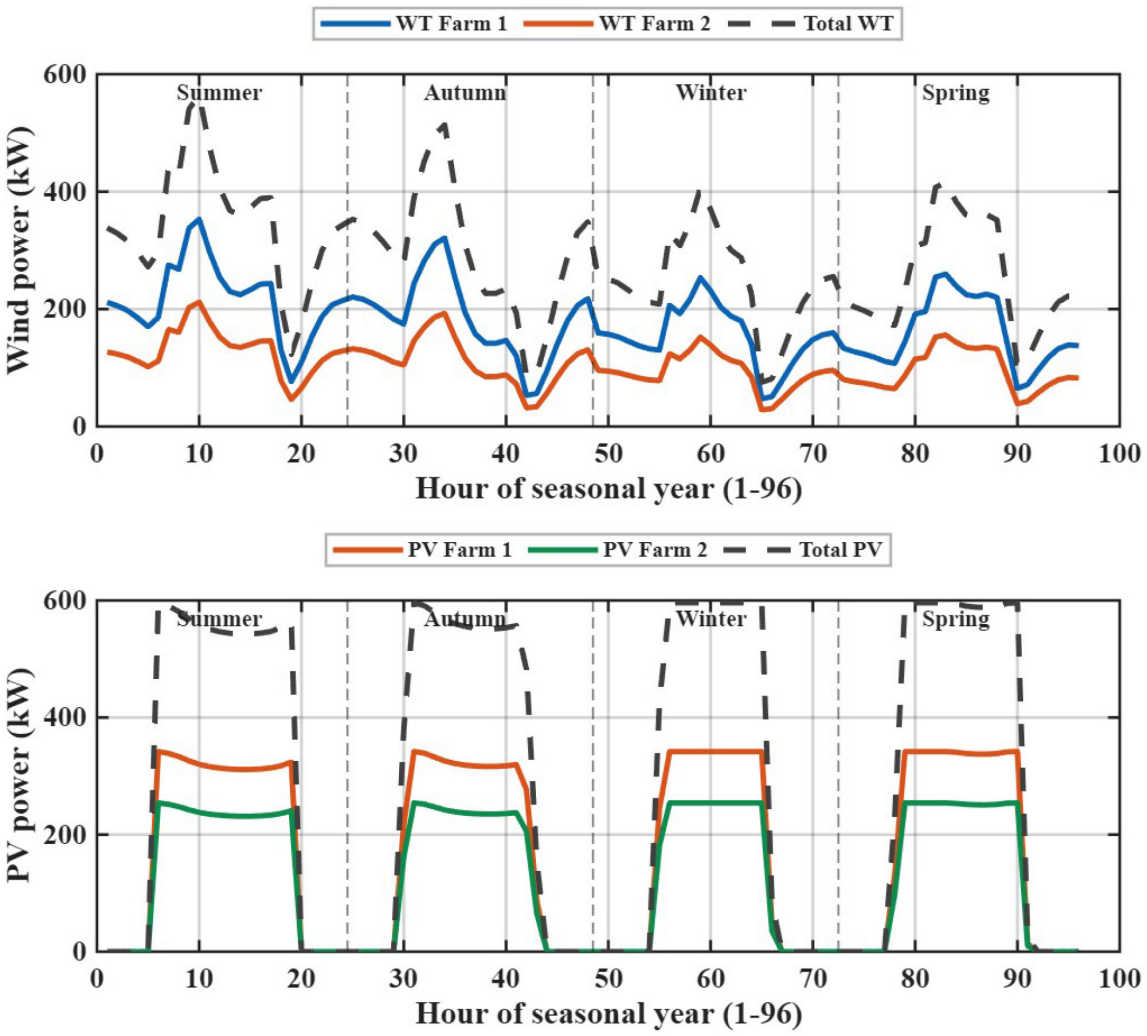
Seasonal WT and PV generation profiles obtained from real weather data and physical conversion models.

The resulting WT and PV generation profiles are presented in Figure 4. Equations 2 and 3 define the uncertainty characterization layer, whereas Equations 5–7 define the deterministic physical conversion layer used to construct the renewable-power target.

### 2.3 ANN renewable-generation estimator

The learning block maps meteorological and temporal features into total renewable generation. The input vector is


$$\begin{array}{l} x\left(t\right)={\left[v\left(t\right),G\left(t\right),T_{a}\left(t\right),h\left(t\right),s\left(t\right)\right]}^{T}, \end{array}$$ (8)

and the ANN prediction is


$$\begin{array}{l} {\hat{P}}_{RES}\left(t\right)=F_{ANN}\left(x\left(t\right);\theta \right), \end{array}$$ (9)

where θ represents trainable weights and biases. The training objective minimizes


$$\begin{array}{l} J_{ANN}=\frac{1}{N}\overset{N}{\underset{t=1}{\sum }}{\left(P_{RES}\left(t\right)-{\hat{P}}_{RES}\left(t\right)\right)}^{2}. \end{array}$$ (10)

**Figure 5:**
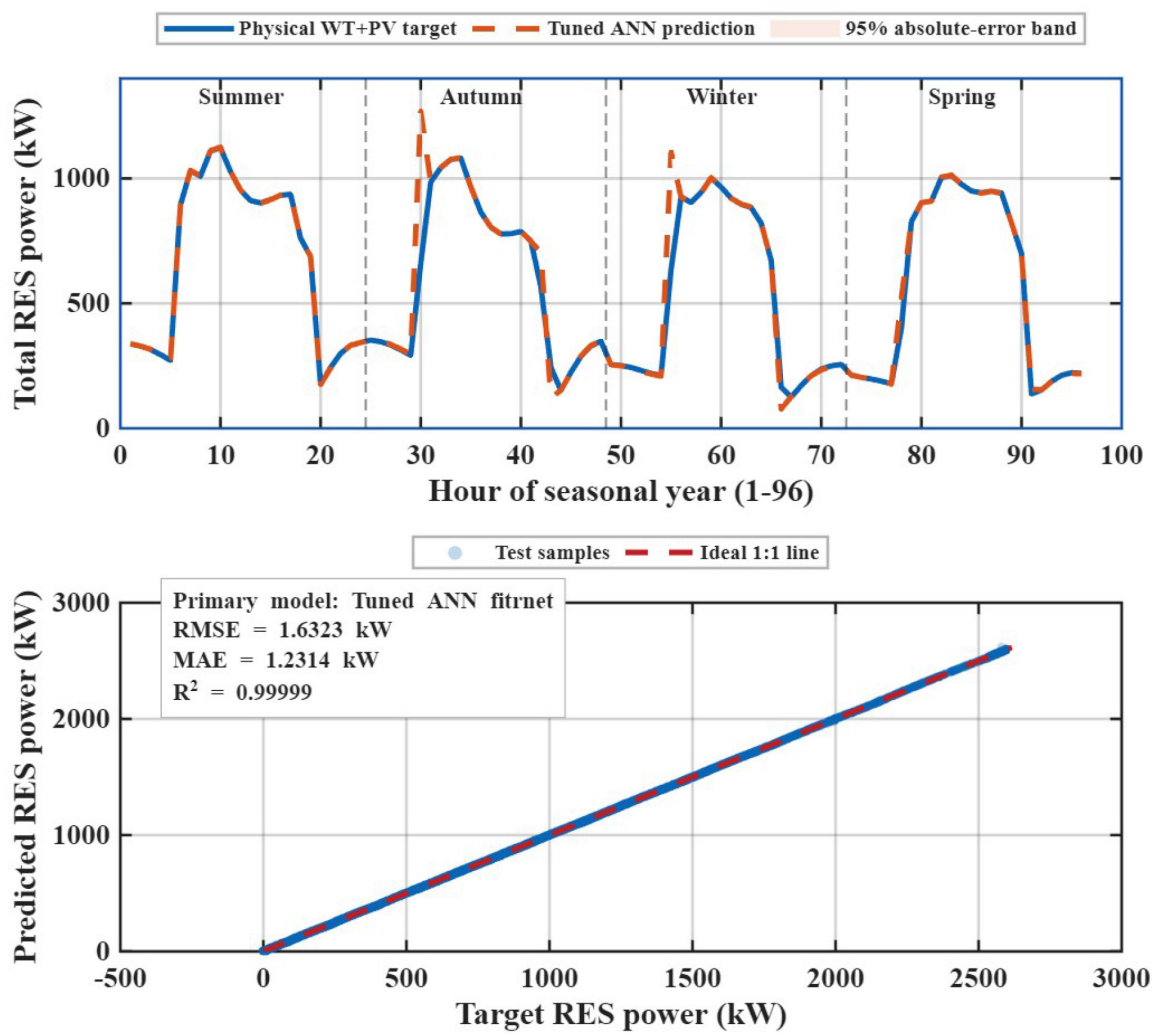
Tuned-ANN renewable-generation performance. The upper panel compares the 96-h physical target with the ANN estimate and its 95% absolute-error band; the lower panel shows the hold-out target–estimate scatter and ideal 1:1 line.

ANN, LSBoost, and Gaussian-process regression are compared as candidate predictors. Figure 5 reports both the seasonal prediction and the actual–predicted scatter, while Table 1 gives the numerical error values. Equations 8–10 specify, respectively, the ANN input vector, the non-linear renewable-power estimate, and the mean-squared-error training objective.

**Table 1:** Learning-model performance used to select the predictive core of the ANN–PSO–VoltVAR framework.

Model	RMSE (kW)	MAE (kW)	*R* ^2^	MAPE (%)	Planning relevance
Tuned ANN fitrnet	1.6323	1.2314	0.99999	2.8027	Best RMSE and MAE; retained as the renewable-generation estimator inside PSO.
LSBoost ensemble	3.1729	2.0182	0.99997	1.7556	Lowest percentage error, but its larger RMSE and MAE make it less suitable for sizing decisions.
Gaussian process	15.2510	1.7423	0.99940	1.8328	Low typical error but the largest RMSE, indicating sensitivity to a small number of large deviations.

The table is built directly in LaTeX, as required by Frontiers.

The selected ANN was implemented in MATLAB using fitrnet. The input features were hub-height wind speed, irradiance, ambient temperature, sine/cosine hour encoding, and sine/cosine month encoding. The response variable was the total WT+PV renewable generation obtained from Equations 5–7. The data were partitioned into 80% training data and an independent 20% hold-out test set. Within the training partition, 15% of the samples were reserved for validation-based tuning, so the hold-out test data were not used for model selection. Four modest hidden-layer configurations, [32, 16], [48, 24], [64, 32, 16], and [48, 24, 12], were examined with ReLU activation, standardized inputs, and *L*_2_ regularization in the range 5 × 10^−5^–2 × 10^−4^. The validation procedure retained the two-layer [32, 16] network, which was refitted on the complete training partition with a maximum of 1,100 iterations. The LSBoost baseline used 350 learning cycles, a learning rate of 0.035, and regression trees with a minimum leaf size of 12. The Gaussian-process baseline used an ARD squared-exponential kernel and a random subset of at most 6,000 training samples to limit computational cost.

The ANN target is generated from the same weather variables through the physical equations; therefore, the ANN is not claimed to introduce new physical information beyond Equations 5–7. Its role is a surrogate and integration layer: It provides a compact weather-to-generation mapping that can be embedded in the optimizer, compared with other regressors, and later recalibrated with measured inverter or turbine data without changing the PSO–VoltVAR planning structure. To avoid any ambiguity, an additional direct-physical-equation ablation is reported in Table 2.

**Table 2:** Mean-profile electrical comparison between the no-RES case, the historical IFLO benchmark, and controlled component-ablation stages.

Case	WT units	PV strings	Volt–VAR (%)	Loss ↓(kW)	VD ↓(p.u.)	*V*_min_↑(p.u.)	VSI ↑(p.u.)	Loss red. (%)
No RES	0	0	0	118.2953	0.30662	0.92520	0.73550	0.0000
IFLO	8	129	–	35.1939	0.06590	0.97040	0.88840	70.2491
Fixed-run sizing only	14	112	0	17.2465	0.05890	0.97554	0.94371	85.4210
Fixed-run direct equation + VV	14	112	33.08	16.3462	0.05231	0.98072	0.95850	86.1827
Fixed-run ANN–PSO–VV	14	112	33.08	15.9888	0.05110	0.98138	0.96096	86.4840
Verified five-start mean-profile solution	12	142	33.25	15.9900	0.051082	0.98213	0.96307	86.4830
Unconstrained gain over historical IFLO	+4	+13	Added	−54.57%	−22.49%	+1.21%	+8.41%	+16.2339 pts

The unequal-capacity rows show absolute planning outcomes and are not a capacity-matched optimizer ranking. Arrows indicate the desired direction of improvement.

The conversion from the ANN total to bus-level injections is explicit. Let $p_{WT}^{u}\left(t\right)$ and $p_{PV}^{s}\left(t\right)$ be the physical output of one WT unit and one PV string. For candidate **X**, the uncorrected contributions are ${\tilde{P}}_{WT,b}=N_{WT,b}p_{WT}^{u}$ and ${\tilde{P}}_{PV,b}=N_{PV,b}p_{PV}^{s}$. A weather-dependent correction factor is


$$\begin{array}{l} \kappa \left(t\right)=\frac{{\hat{P}}_{RES}^{ref}\left(t\right)}{\max\left(\overset{ref}{\underset{RES,phys}{P}}\left(t\right),\varepsilon \right)}. \end{array}$$ (11)

The four injections are


$$\begin{array}{l} P_{WT,b}\left(t\right)=\kappa \left(t\right){\tilde{P}}_{WT,b}\left(t\right),\;P_{PV,b}\left(t\right)=\kappa \left(t\right){\tilde{P}}_{PV,b}\left(t\right), \end{array}$$ (12)

so their sum is exactly the candidate renewable total. Thus, the ANN modifies weather-dependent availability, while the PSO capacity variables determine the WT/PV and bus shares.

### 2.4 ANN–PSO–VoltVAR capacity-allocation formulation

**Figure 6:**
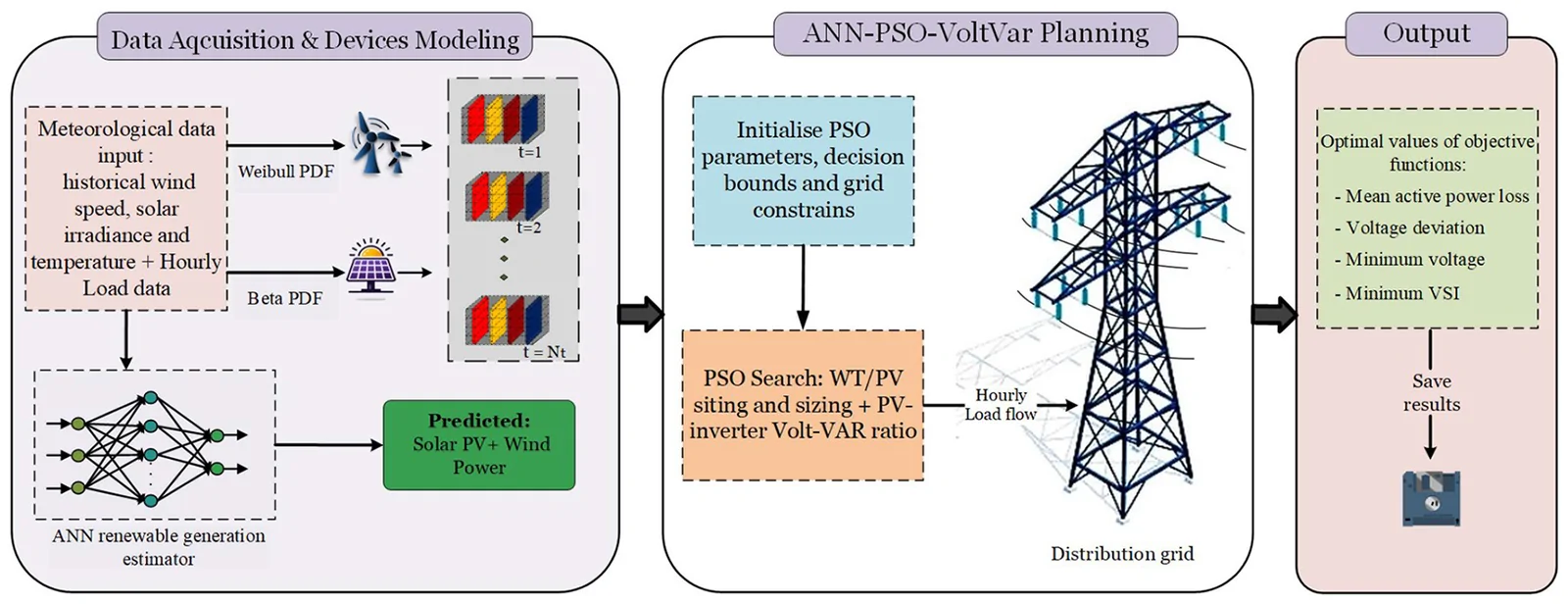
Developed renewable generation model combining real meteorological data, WT/PV physical models, and ANN-based renewable generation estimation. The figure shows the central data-to-generation chain.

The proposed workflow is summarized in Algorithm 1; the weather-to-generation chain is detailed in Figure 6. The four buses are preselected candidates and are not location decision variables. The optimization vector is


$$\begin{array}{l} X=\left[N_{WT,35},N_{WT,68},N_{PV,52},N_{PV,70},\eta_{52},\eta_{70}\right]. \end{array}$$ (13)

Algorithm 1 1:  Read hourly wind speed, irradiance, temperature and load data; construct the normalized load using Equation 1.
2:  Fit the Weibull and Beta distributions using Equations 2 and 3, then build the common meteorological scenarios used in Equation 4.
3:  Compute the physical WT and PV unit profiles using Equations 5–7.
4:  Train and validate the ANN using Equations 8–10; obtain the renewable estimate and bus-allocation correction through Equations 11 and 12.
5:  Initialize the particle population from the decision vector in Equation 13; set iteration counter *r*←1.
6:  **while** *r* ≤ *r*_max_ **do**
7:    **for** each particle *p* **do**
8:       **for** each meteorological scenario *s* **do**
9:          Decode integer WT/PV capacities and continuous Volt–VAR limits.
10:          Compute the bus-level renewable injections using Equation 12.
11:         **while** the voltage-dependent reactive-power update has not converged **do**
12:           **Evaluate** the Volt–VAR command from Equation 14 and enforce inverter capability using Equation 15.
13:           Recalculate loss, voltage deviation, minimum voltage and VSI using Equations 16–18.
14:           **if** a voltage, VSI, inverter-capability or curtailment limit is violated **then**
15:               Add the corresponding penalty term to the scenario objective in Equation 19.
16:           **end if**
17:         **end while**
18:       **end for**
19:       Aggregate the scenario costs with the expected-cost/CVaR objective in Equation 4.
20:       **if** the current cost improves the personal best **then**
21:          Update **pbest**_*p*_.
22:       **end if**
23:       **if** the current cost improves the global best **then**
24:          Update **gbest**.
25:       **end if**
26:    **end for**
27:    Update particle velocity and position using Equations 20 and 21; round the capacity variables and bound all decision variables.
28:    *r*←*r*+1.
29:   **end while**
30:  Return the best allocation, inverter settings and electrical indicators.

The first four variables are integer capacity-allocation variables, and η_*i*_∈[0, 0.35] is the maximum reactive-support fraction of PV inverter *i*. Therefore, this formulation optimizes sizing and allocation at predetermined buses, not network-wide siting.

Reactive power is governed by a voltage-dependent Volt–VAR curve rather than a fixed ratio. With breakpoints *V*_1_ = 0.94, *V*_2_ = 0.98, *V*_3_ = 1.02, and *V*_4_ = 1.06 p.u., the normalized droop function is


$$\begin{array}{l} \psi \left(V\right)=\{\begin{array}{ll} 1, & V\leq V_{1},\\ \left(V_{2}-V\right)/\left(V_{2}-V_{1}\right), & V_{1}<V<V_{2},\\ 0, & V_{2}\leq V\leq V_{3},\\ -\left(V-V_{3}\right)/\left(V_{4}-V_{3}\right), & V_{3}<V<V_{4},\\ -1, & V\geq V_{4}. \end{array} \end{array}$$ (14)

The requested command is $Q_{i}^{req}\left(t\right)=\eta_{i}S_{inv,i}\psi \left(V_{i}\left(t\right)\right)$. With inverter loading ratio 1.10 and minimum power factor 0.90, the accepted command satisfies


$$\begin{array}{l} |Q_{i}\left(t\right)|\leq \\ \min\left(\eta_{i}S_{inv,i},\sqrt{S_{inv,i}^{2}-P_{PV,i}{\left(t\right)}^{2}},P_{PV,i}\left(t\right)\tan\left(\cos^{-1}\left(0.90\right)\right)\right), \end{array}$$ (15)

where $S_{inv,i}=1.10P_{PV,i}^{rated}$. If *P*_*PV, i*_>*S*_*inv, i*_, the excess is recorded as active-power curtailment before the reactive command is applied.

The active loss is evaluated in the calibrated branch-response form


$$\begin{array}{l} P_{loss}=\overset{N_{b}-1}{\underset{\ell =1}{\sum }}R_{\ell }\frac{P_{\ell }^{2}+Q_{\ell }^{2}}{|V_{\ell }{|}^{2}}, \end{array}$$ (16)

while the time-series voltage deviation and minimum voltage are defined consistently as


$$VD=\frac{1}{TN_{b}}\overset{t=1}{\underset{T}{\sum }}\overset{i=1}{\underset{N_{b}}{\sum }}\left|V_{i}(t)-1\right|,V_{\min}=\underset{t,i}{\min}\left|V_{i}(t)\right|.$$ (17)

The radial-feeder stability indicator is


$$\begin{array}{l} VSI_{j}=|V_{i}{|}^{4}-4{\left(P_{j}X_{ij}-Q_{j}R_{ij}\right)}^{2}-4\left(P_{j}R_{ij}+Q_{j}X_{ij}\right)|V_{i}{|}^{2}. \end{array}$$ (18)

For scenario *s*, the constrained cost is


$$\begin{array}{lll} F_{s}\left(X\right) & = & w_{1}\frac{P_{loss,s}}{P_{loss}^{base}}+w_{2}\frac{VD_{s}}{VD^{base}}+w_{3}\Pi_{V,s}+w_{4}\Pi_{VSI,s}+w_{5}\Pi_{inv,s} \end{array}$$

$$\begin{array}{ll} + & w_{6}\Pi_{curt,s}, \end{array}$$ (19)

with voltage, stability, inverter capability, and curtailment penalties. Equation 4 aggregates these scenario costs. The reference thresholds are selected from the IFLO benchmark, forcing the search to improve voltage and stability rather than minimizing loss alone. Particle velocity and position are updated as


$$\begin{array}{l} u_{p}^{r+1}=\omega u_{p}^{r}+c_{1}r_{1}\left({pbest}_{p}-x_{p}^{r}\right)+c_{2}r_{2}\left(gbest-x_{p}^{r}\right), \end{array}$$ (20)

$$\begin{array}{l} x_{p}^{r+1}=x_{p}^{r}+u_{p}^{r+1}. \end{array}$$ (21)

WT and PV sizing variables are rounded to the nearest integer after each update, while Volt–VAR ratios remain continuous.

The PSO settings are explicitly stated for reproducibility. Each run uses 50 particles and 120 iterations, with *c*_1_ = *c*_2_ = 1.55, inertia decreasing linearly from 0.90 to 0.40, and velocity limited to 20% of each variable range. Twenty independent starts use seeds 42–61. For each run, the code records the robust objective, decision vector, scenario-mean loss, scenario-mean voltage deviation, worst-scenario minimum voltage, worst-scenario minimum VSI, curtailment fraction, capability violation, and elapsed time. It exports both run-level data and the mean, standard deviation, and best and worst values. The lowest-cost run is subjected to deterministic coordinate refinement. A capacity-matched controlled mode fixes the total WT and PV capacities to the IFLO totals and applies identical scenarios, constraints, objective weights, and PSO evaluation budgets. It re-evaluates the historical IFLO allocation and optimizes the capacity split and inverter settings, but it is not presented as a reimplementation of the unavailable original IFLO optimizer.

## 3 Results

### 3.1 Prediction performance and optimization convergence

**Figure 7:**
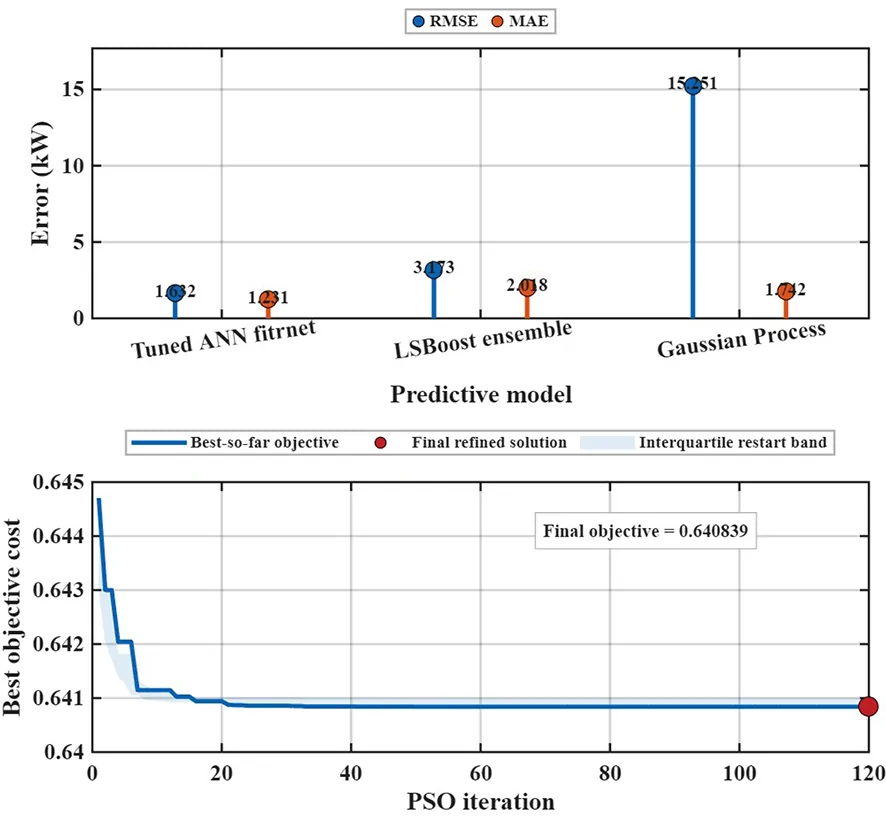
Learning and optimization behavior. The upper panel compares RMSE and MAE for the three regressors. The lower panel shows the convergence trace retained from the verified mean-profile optimization. Electrical benchmark values are taken from Table 2; the revised twenty-run scenario statistics are generated separately when the supplementary code is executed.

The tuned ANN produced the best absolute-error performance, with RMSE = 1.6323 kW, MAE = 1.2314 kW, and *R*^2^ = 0.99999 (Table 1). LSBoost achieved the lowest MAPE (1.7556%) but larger RMSE and MAE values of 3.1729 and 2.0182 kW, respectively. Because percentage errors can be disproportionately affected when the renewable target is small, the model choice was based primarily on RMSE and MAE, which measure the injection error directly in kW. The Gaussian-process model yielded an MAE of 1.7423 kW and an RMSE of 15.251 kW; this large RMSE–MAE separation is consistent with a limited number of comparatively large errors, which is undesirable when renewable-injection errors propagate into sizing decisions. Figure 5 confirms the close agreement between the tuned ANN and the physical renewable target and displays the 95% absolute-error band. Figure 7 links model selection to the verified mean-profile convergence trace: Most of the objective decrease occurs within the early iterations, after which the retained run stabilizes at 0.640839. The scenario-based twenty-run outputs are generated separately by the supplementary code and are not inferred from this legacy convergence panel.

The optimized solution was


$$\begin{array}{l} X^{⋆}=\left[6,6,71,71,33.25,33.25\right], \end{array}$$ (22)

**Figure 8:**
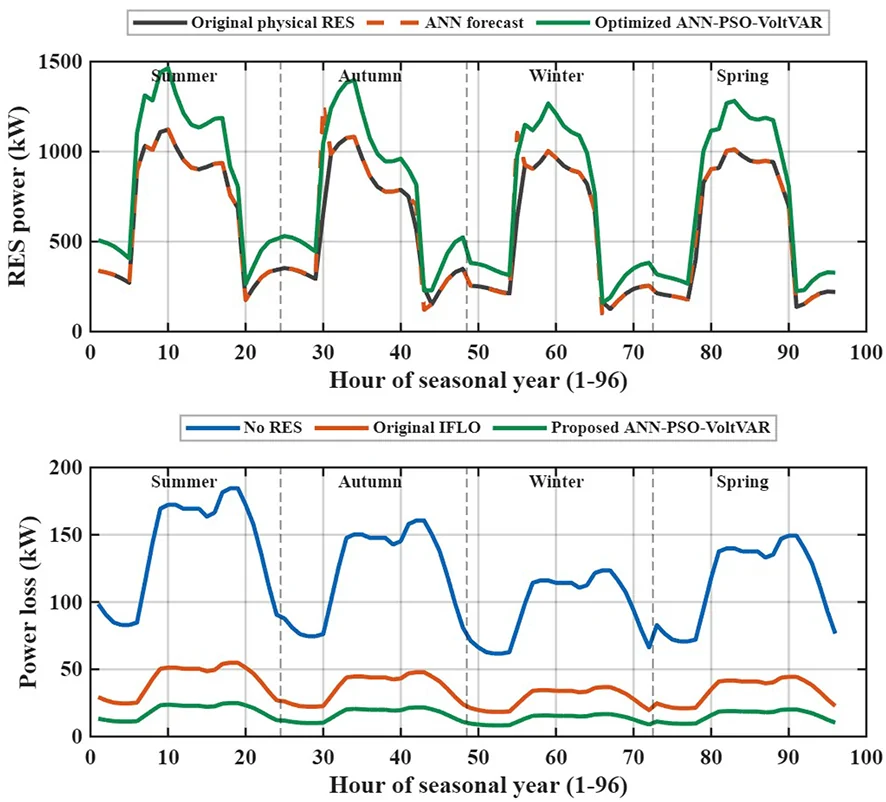
Verified mean-profile ANN–PSO–VoltVAR renewable generation and seasonal loss profiles. The proposed configuration maintains lower loss than the no-RES and IFLO cases across the four representative seasonal periods.

The optimized decision vector reported in Equation 22 corresponds to 6 WTs at bus 35, 6 WTs at bus 68, 71 PV strings at bus 52, 71 PV strings at bus 70, and approximately 33.25% Volt–VAR support from each PV inverter. The optimized renewable generation and corresponding seasonal loss profiles are shown in Figure 8.

### 3.2 Benchmark comparison

Table 2 compares the no-RES case, the IFLO benchmark, the fixed-inertia ablation stages, and the verified five-start mean-profile ANN–PSO–VoltVAR configuration. The verified five-start mean-profile configuration reduced mean active loss to 15.9900 kW, lowered voltage deviation to 0.051082 p.u., increased minimum voltage to 0.98213 p.u., and raised minimum VSI to 0.96307. Relative to IFLO, these values correspond to a 54.57% reduction in residual mean loss, a 22.49% reduction in voltage deviation, a 1.21% increase in minimum voltage, and an 8.41% increase in minimum VSI. The four indicators reported in Table 2 move in the desired direction, showing that the verified mean-profile solution does not exchange voltage security for loss reduction.

**Figure 9:**
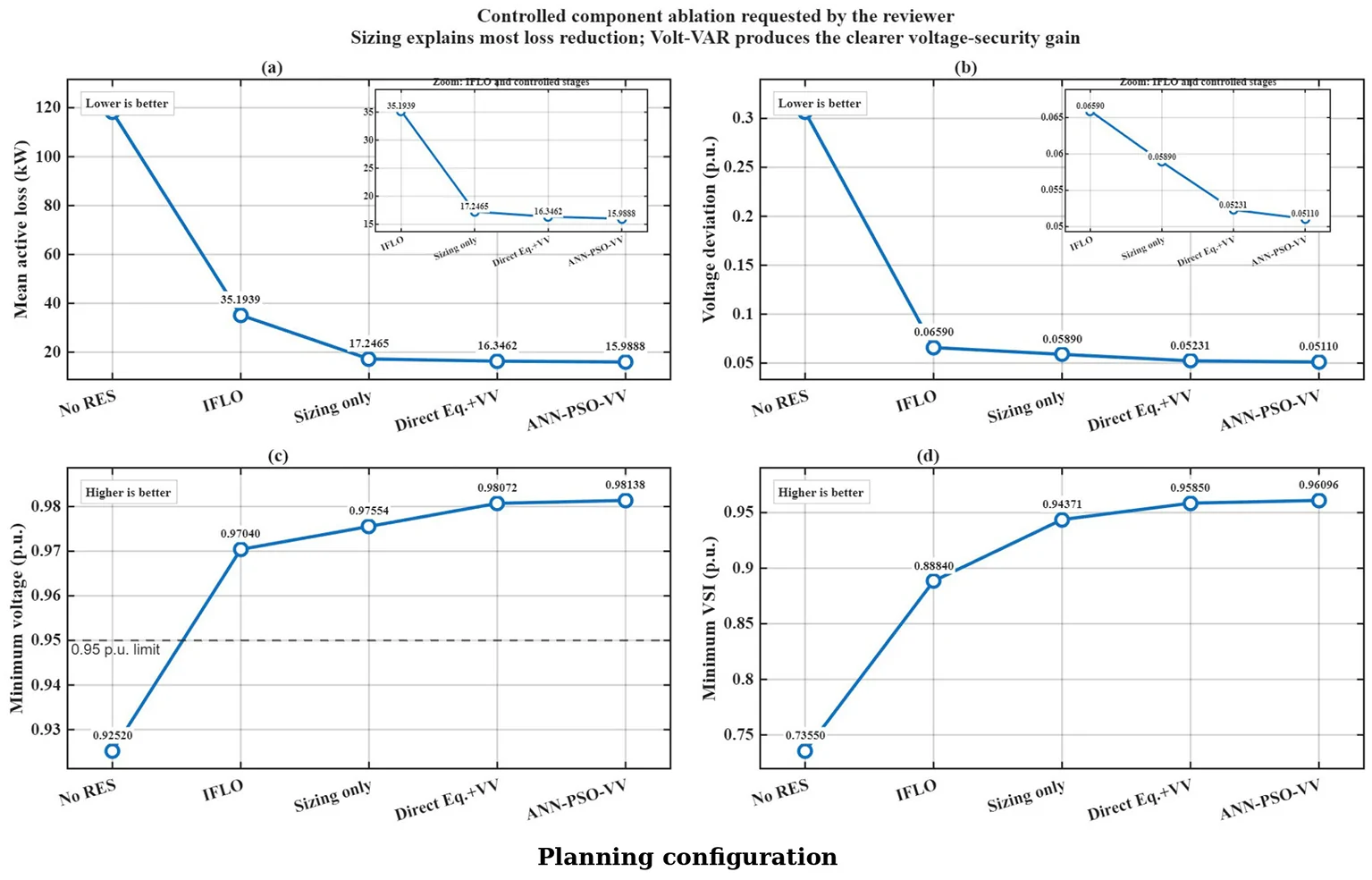
Controlled component ablation requested by the reviewer. The panels report mean active-power loss, mean voltage deviation, minimum bus voltage, and minimum voltage-stability index for the no-RES, IFLO, sizing-only, direct physical-equation PSO–VoltVAR, and ANN-assisted PSO–VoltVAR cases. The zoom panels expose the post-IFLO differences. The figure is a mean-profile mechanism analysis: Sizing explains most of the residual-loss reduction, whereas Volt–VAR support produces the clearer voltage-security gain. Because installed capacities differ between some rows, it is not presented as a fair optimizer ranking. **(a)** Mean active-power loss. **(b)** Mean voltage deviation. **(c)** Minimum bus voltage. **(d)** Minimum voltage-stability index.

**Figure 10:**
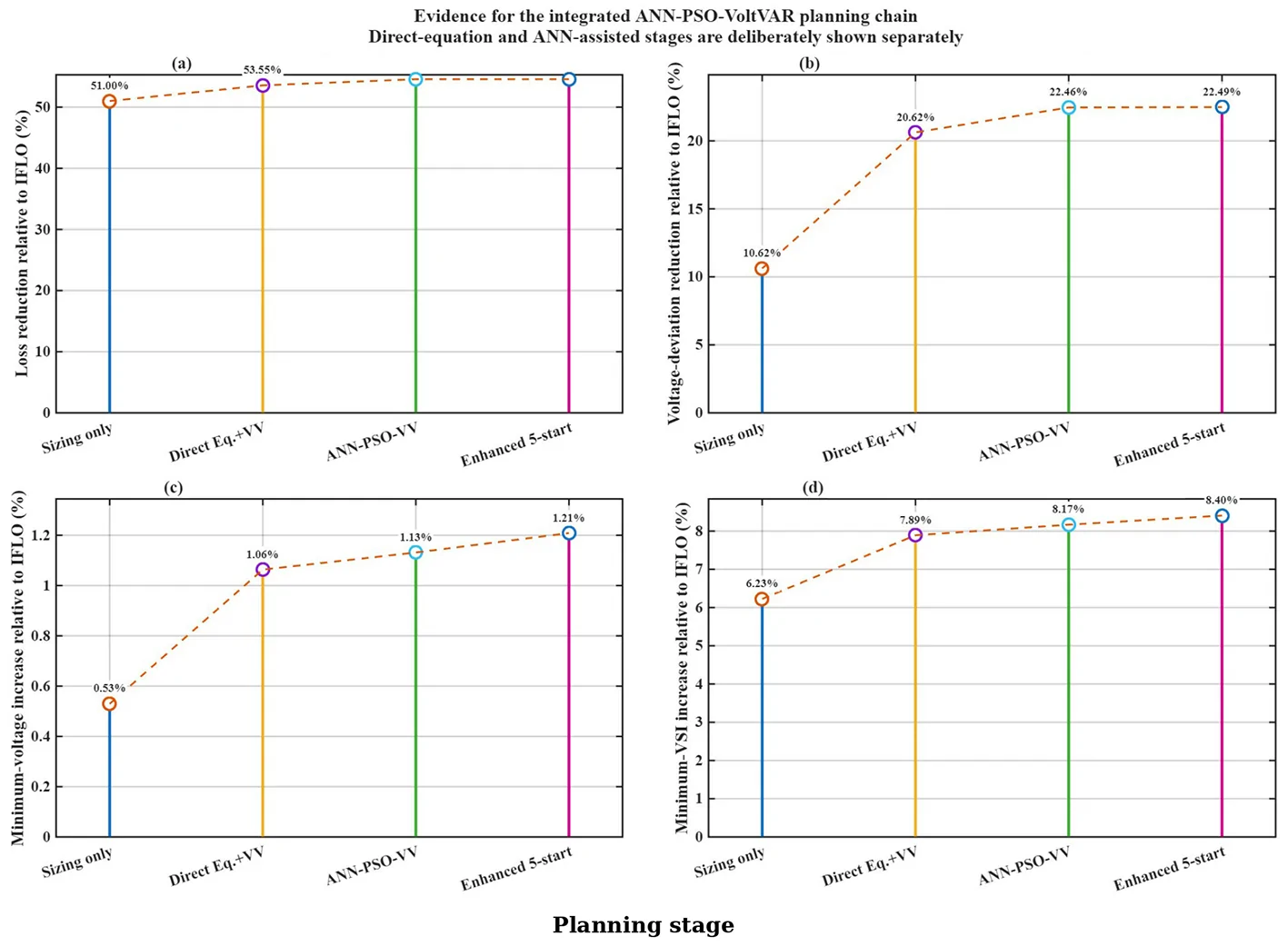
Normalized mean-profile evidence for the integrated ANN–PSO–VoltVAR planning chain. Every post-IFLO stage is expressed as a percentage improvement relative to the historical IFLO result; unequal-capacity stages are not interpreted as a capacity-matched optimizer comparison. The direct-equation and ANN-assisted stages are deliberately separated to test the ANN-surrogate question, while the verified five-start mean-profile solution distinguishes component ablation from the earlier retained final point. The scenario-based twenty-run results are generated separately by the supplementary code and are not inferred from this mechanism figure. **(a)** Active loss contribution by planning stage. **(b)** Voltage-deviation contribution by planning stage. **(c)** Voltage-floor contribution by planning stage. **(d)** Stability margin contribution by planning stage.

The historical IFLO and unconstrained proposed rows have different installed capacities and are therefore interpreted as absolute planning outcomes, not as a pure optimizer contest. The supplementary code provides a controlled capacity-matched mode that fixes the totals at 8 WT units and 129 PV strings and applies the same meteorological scenarios, calibrated electrical-response equations, inverter constraints, objective weights, and PSO evaluation budget. The historical IFLO allocation is re-evaluated under these common conditions, while PSO optimizes only the capacity split and inverter settings. Because the original IFLO source code is unavailable, this is a capacity-matched plan comparison rather than an algorithm-to-algorithm reproduction. The mean-profile Figures 9 and 10 are used only to explain component mechanisms. The revised code generates twenty-run detailed and summary files; hardware-dependent computation times and the resulting statistics must be regenerated on the authors' final machine and reported with the processor, memory, operating system, and MATLAB release.

The mechanism behind these improvements is electrical rather than purely algorithmic. The sizing variables reduce branch current by moving part of the active-power supply closer to the load centers, which explains the reduction in active losses. The Volt–VAR variables then provide a local reactive-power actuator at the PV buses, reducing voltage deviation and lifting the voltage floor without requiring additional PV oversizing. This is why the proposed case improves loss, *VD*, *V*_min_, and VSI simultaneously rather than improving one metric at the expense of another.

To establish that the improvement is not produced by PSO alone, Table 2 preserves the controlled fixed-inertia single-start ablation. Its sizing-only case removes Volt–VAR support, whereas its direct-equation case retains Volt–VAR support but bypasses the ANN. These stages isolate the contributions of active-power sizing, inverter reactive support, and the ANN surrogate. A separate final row reports the verified five-start mean-profile solution, preventing the earlier ablation configuration from being mistaken for the enhanced optimum.

Figure 9 displays the controlled fixed-inertia sequence. The sizing-only stage decreases mean active loss from 35.1939 kW for IFLO to 17.2465 kW, but its voltage deviation, minimum voltage, and minimum VSI remain at 0.05890 p.u., 0.97554 p.u., and 0.94371. Adding Volt–VAR support to direct physical-equation evaluation improves these values to 0.05231 p.u., 0.98072 p.u., and 0.95850, while reducing mean loss to 16.3462 kW. The corresponding ANN-assisted fixed-inertia stage reaches 15.9888 kW, 0.05110 p.u., 0.98138 p.u., and 0.96096. Thus, source sizing accounts for most of the loss decrease, while inverter support produces the clearer voltage quality and stability gains. The small difference between the direct-equation and ANN-assisted stages supports the stated role of the ANN as a compact surrogate rather than an additional source of physical information. The verified five-start mean-profile result in Table 2 has an effectively unchanged mean loss of 15.9900 kW (a difference of only 0.0012 kW from the controlled ANN-assisted stage), while further raising minimum voltage and VSI to 0.98213 and 0.96307. This is consistent with the multi-objective formulation: Once loss is already low, the search can favor a design with a stronger voltage-security margin rather than minimizing loss alone.

Figure 10 complements the absolute-value ablation by expressing each post-IFLO planning stage as a percentage improvement relative to IFLO. The sizing-only stage accounts for a 51.00% reduction in residual mean loss, but only a 10.62% reduction in voltage deviation, a 0.53% increase in minimum voltage, and a 6.23% increase in minimum VSI. Adding Volt–VAR support with direct physical-equation evaluation raises the corresponding gains to 53.55%, 20.62%, 1.06%, and 7.89%. The ANN-assisted stage reaches 54.57%, 22.46%, 1.13%, and 8.17%, respectively, while the verified five-start mean-profile solution preserves the 54.57% loss improvement and increases the minimum-voltage and VSI gains to 1.21% and 8.40%. These normalized trends substantiate the integrated-chain claim: Source sizing dominates the active-loss decrease, inverter reactive support produces the clearest additional voltage-security gain, and the ANN acts as a compact surrogate rather than as an artificial source of physical information.

### 3.3 Voltage profile and stability behavior

**Figure 11:**
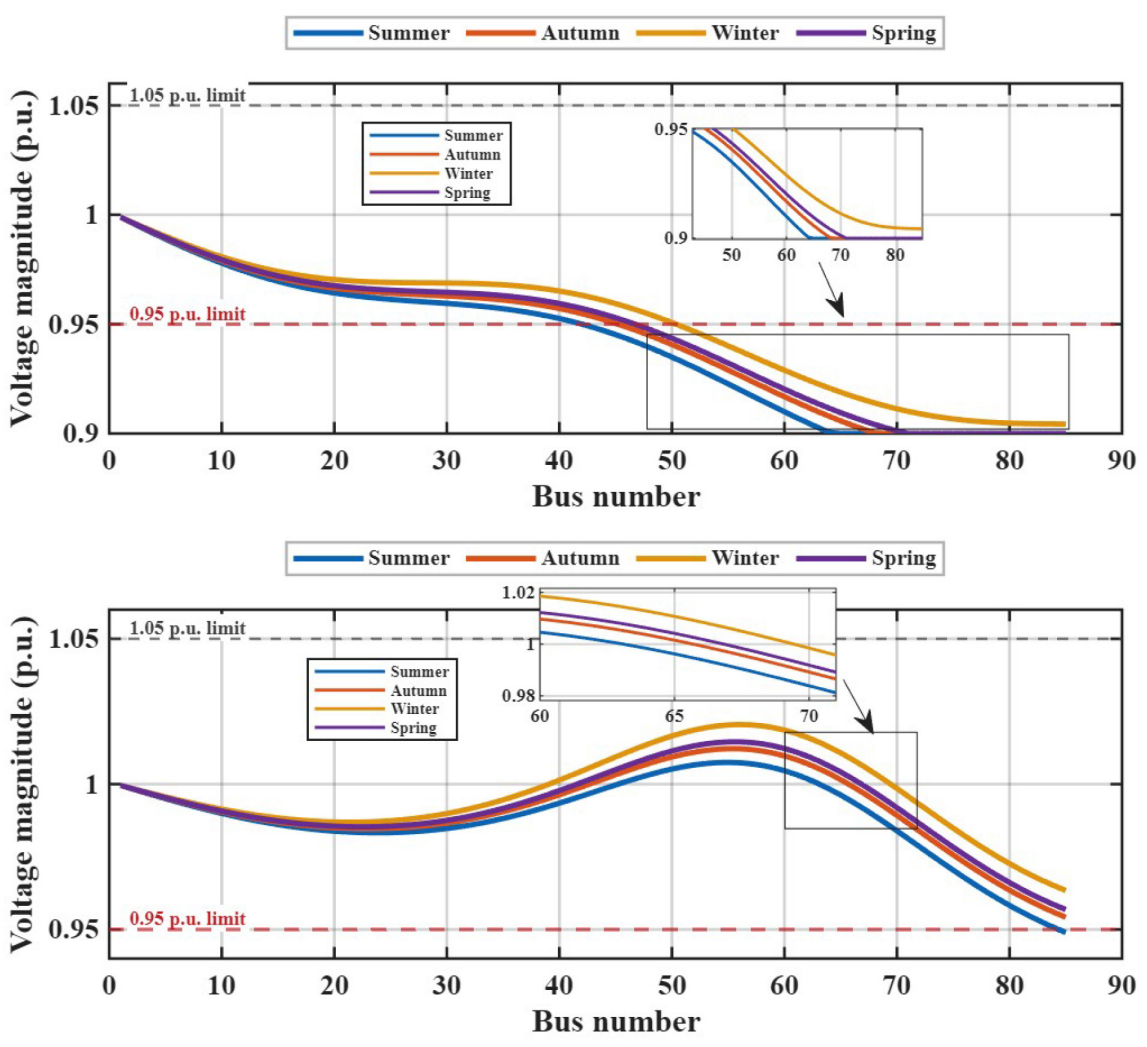
Seasonal voltage profiles without RES and with the IFLO benchmark. The comparison shows the voltage recovery achieved by the benchmark before adding Volt–VAR support.

**Figure 12:**
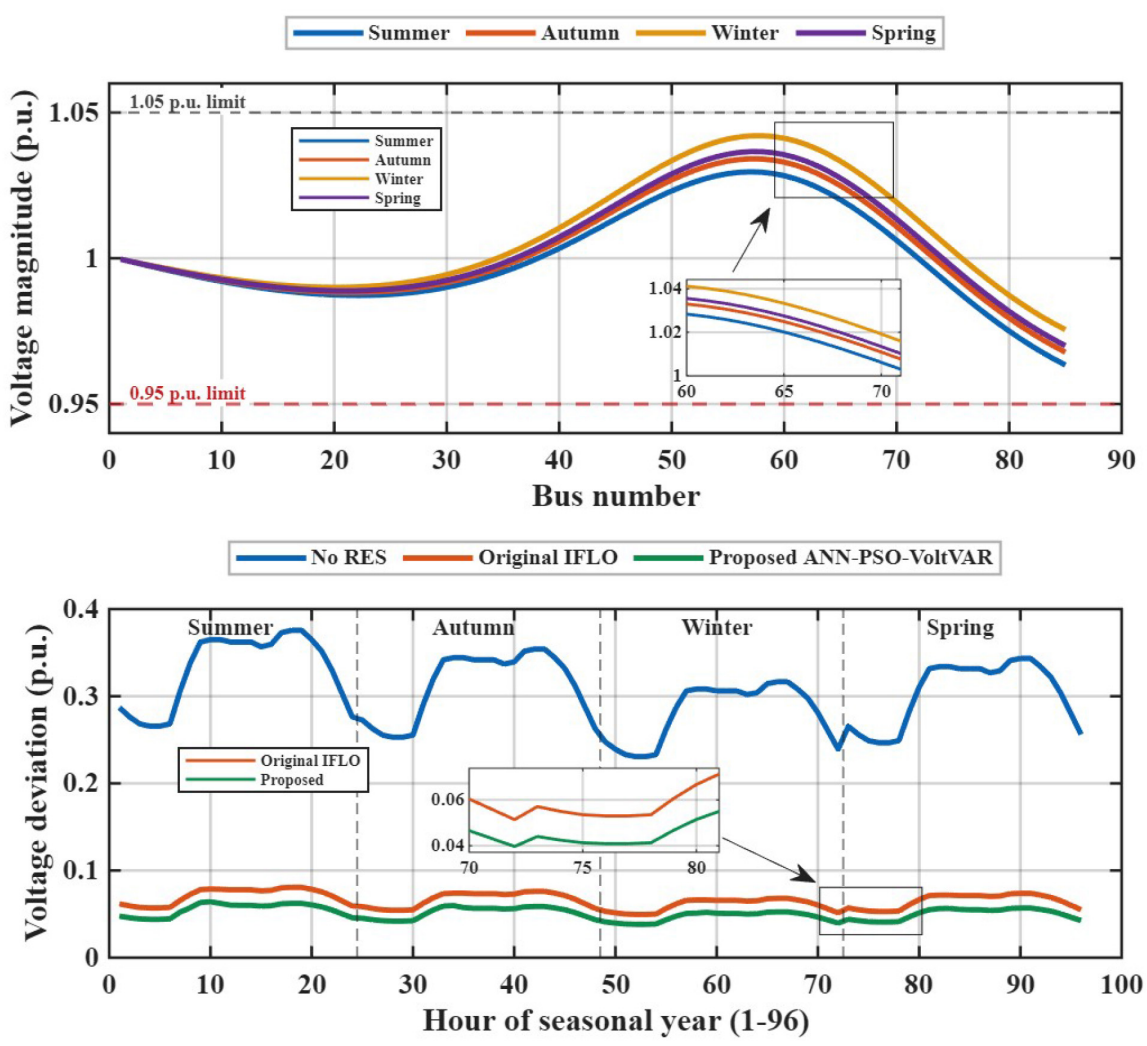
Voltage profile and voltage deviation under the proposed ANN–PSO–VoltVAR solution. The two-panel figure highlights the voltage-floor reinforcement obtained through smart-inverter support.

**Figure 13:**
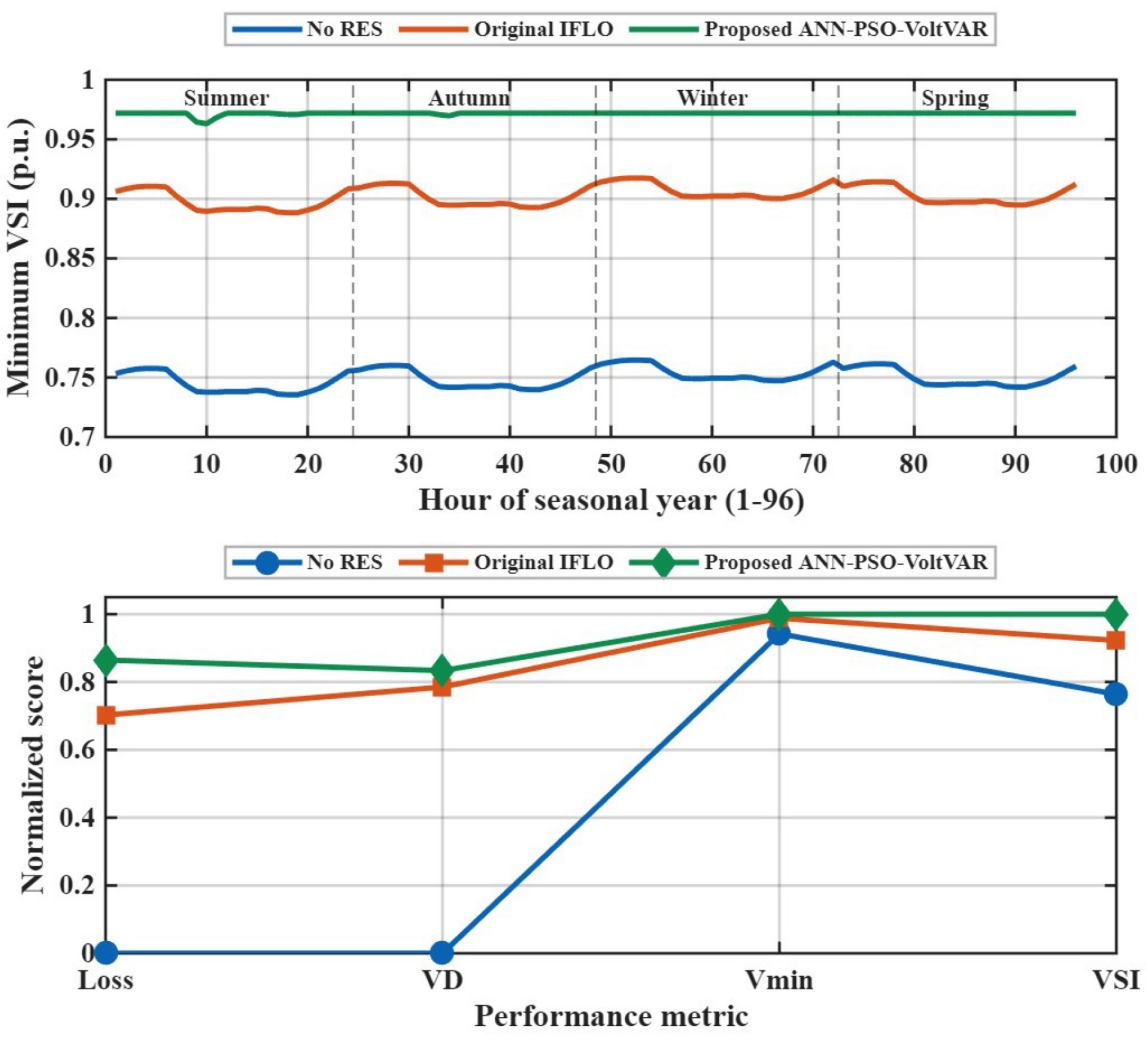
Minimum VSI profiles and normalized multi-metric summary. The verified five-start mean-profile solution raises minimum VSI to 0.96307 while retaining the reductions in mean loss and voltage deviation.

The voltage-profile results in Figures 11 and 12 show the physical meaning of the numerical gains. Without RES, the radial feeder operates near a weak voltage band. The IFLO solution improves the voltage profile but still leaves a stability margin that can be exploited. The ANN–PSO–VoltVAR solution lifts the voltage floor while reducing voltage deviation, because PV inverters are no longer treated as passive active-power injectors. The minimum VSI profile and the compact multi-metric summary in Figure 13 confirm that the stability margin moves away from the fragile low-stability region.

## 4 Discussion

The results support a strict conclusion: The improvement is not a consequence of replacing IFLO by PSO alone. If the method were only a different optimizer, the result would be less convincing because metaheuristic comparisons can be sensitive to tuning, stopping criteria and calibration assumptions. The stronger contribution is the closed planning chain. The ANN learns weather-to-generation behavior from real data; PSO searches a mixed discrete–continuous planning vector; and Volt–VAR support provides a physically meaningful voltage-control actuator. This explains why the method simultaneously reduces loss, reduces VD, raises *V*_*min*_, and improves VSI.

The staged trends in Figures 9 and 10 reinforce this conclusion. The absolute-value ablation shows the electrical indicators attained by each controlled case, whereas the normalized contribution figure makes the mechanism easier to interpret: The dominant loss decrease occurs after source sizing, while the transition from sizing only to Volt–VAR-enabled planning produces the clearest additional gains in voltage deviation, minimum voltage, and VSI. The close direct-equation and ANN-assisted results also confirm that the ANN is used as a surrogate interface rather than being credited with new physical information.

The comparison also changes the technical reading of renewable sizing. The IFLO benchmark used 8 WT units and 129 PV strings, whereas the enhanced configuration uses 12 WT units and 142 PV strings with 33.25% inverter support. Under the selected In Salah meteorological conditions and calibrated IEEE 85-bus response, the multi-start search therefore favors a balanced 6/6 WT allocation together with a symmetric 71/71 PV allocation and nearly symmetric inverter support. The Volt–VAR decision addresses the typical weakness of active-power-only planning: raising the voltage floor and strengthening weak buses without relying solely on source oversizing.

The comparison with the four added studies clarifies the novelty boundary in a more precise way. The hybrid EV-charging studies of Soomro et al. (2024) and Kumar et al. (2024) are closest to the renewable-resource part of the present study because they show how PV, wind, and hydrogen-based subsystems can be sized under energy and economic constraints. Their focus, however, remains the stand-alone or off-grid energy station, while the present study places renewable sizing inside a radial feeder where voltage deviation, minimum voltage, and VSI are part of the optimization assessment.

The distribution-network studies of Memon et al. (2023) and Memon et al. (2022) are closer to the feeder-performance part of the present study because they address loss reduction, DG allocation, and network technical indicators. Their contribution supports the importance of optimized DG placement, but they do not combine a learned meteorological generation surrogate with mixed WT/PV sizing and continuous PV-inverter Volt–VAR variables. Therefore, the present manuscript is positioned as an integrated planning-chain contribution rather than as a claim of novelty for Weibull modeling, Beta modeling, ANN regression, or PSO alone.

The study has an important limitation. The IEEE 85-bus electrical results are generated by a benchmark-calibrated response model, not by an independent time-series reconstruction of every branch current and bus voltage. They must therefore be interpreted as a controlled proof-of-concept. A separate exact backward/forward-sweep module is supplied for the complete standard 33-bus feeder; its no-DG base case reproduces approximately 202.677 kW active loss and 0.91309 p.u. minimum voltage at bus 18. This verifies the full-feeder solver but does not retrospectively validate the calibrated IEEE 85-bus values. Direct validation on complete IEEE 85-bus branch/load data and additional networks remains necessary.

## 5 Conclusion

This study presented an ANN–PSO–VoltVAR framework for scenario-based renewable capacity allocation at four preselected buses. The revised formulation provides explicit bus-level WT/PV injections, a voltage-dependent inverter capability curve, meteorological scenarios, a robust expected-cost/CVaR objective, a capacity-matched controlled mode, and a twenty-run statistical protocol. The supplied mean-profile ablation suggests that capacity allocation produces most of the loss reduction, while inverter reactive support produces the clearer additional voltage-security improvement. These observations remain subject to the principal limitation that the IEEE 85-bus electrical response is benchmark-calibrated. The framework should therefore be regarded as a reproducible proof-of-concept, not as a complete IEEE 85-bus validation. The exact 33-bus load-flow module verifies the independent solver implementation, but direct full-feeder validation on the IEEE 85-bus system and other networks is required before broader performance claims are made.

## Data Availability

The datasets presented in this study can be found in online repositories. The names of the repository/repositories and accession number(s) can be found in the article/Supplementary material.
