# Supplementary material for: AI-driven ANN–PSO smart-inverter capacity allocation at preselected buses under meteorological uncertainty
Source: Front Artif Intell. 2026 Aug 20;9:1896551. doi: 10.3389/frai.2026.1896551 (PMC13538838; doi:10.3389/frai.2026.1896551)
Supplement: Supplementary file 1 [file Supplementary_File_2.pdf]

Supplementary Method Note

ANN-PSO-VoltVAR renewable planning under meteorological uncertainty

Selman Djeffal[1], Badreddine Bendriss[2], Abdelhamid Ghoul[3,*], Mohamed Razi Morakchi[4]

Companion to Supplementary Code 1 and Supplementary Data Sheet 1

Purpose. This note documents the reproducibility logic of the MATLAB workflow supplied for production. It explains how the weather data, seasonal profiles, renewable-generation models, ANN/ML predictor, PSO sizing variables, smart-inverter Volt-VAR variables, robust uncertainty layer, and validation outputs are connected. It also distinguishes the verified mean-profile ablation values from the separate scenario-based twenty-run robustness study.

1. Supplementary package and file roles

Production label	File name	Role
Supplementary Code 1	Supplementary_Code_1_ANN_PSO_VoltVAR.m	Executable MATLAB implementation; downloads/loads weather data, builds profiles, trains models, runs optimization, generates figures and exports the consolidated workbook.
Supplementary Data Sheet 1	Supplementary_Data_Sheet_1.xlsx	Seasonal-profile construction, inputs, ANN/ML metrics, verified mean-profile results, decision vectors, robustness settings, validation notes and output-file map.
Supplementary Method Note	Supplementary_Method_Note.pdf	Methodological explanation and reproducibility guide for the two files above.

2. Study case, weather data and seasonal representation

Study site. In Salah, Algeria (latitude 27.1935, longitude 2.46069). The MATLAB script requests hourly NASA POWER data from 1 January 2012 through 31 December 2021 using ALLSKY_SFC_SW_DWN (solar irradiance), WS10M (10-m wind speed) and T2M (ambient temperature). Missing values and nonphysical negative weather entries are removed before profile construction.

Seasonal aggregation. The cleaned hourly data are grouped by season and hour of day. Four representative 24-hour blocks are concatenated in the implementation order Summer, Autumn, Winter and Spring, producing a 96-hour operating horizon. The load profile starts from the 24-hour percentage vector reported in Supplementary Code 1 and applies seasonal factors 1.00, 0.92, 0.78 and 0.88, respectively, before normalization to 100% annual peak. The complete 96-hour load construction is tabulated in Supplementary Data Sheet 1.

Season	Months in code	Load factor
Summer	May-Jul	1.00
Autumn	Aug-Oct	0.92
Winter	Nov-Jan	0.78
Spring	Feb-Apr	0.88

3. Renewable-generation and uncertainty models

Wind model. The 10-m wind speed is extrapolated to the 50-m hub height using v_hub = v_10 (50/10)^(1/7). A 250-kW turbine is modeled with cut-in, rated and cut-out speeds of 3, 12 and 25 m/s. Output between cut-in and rated speed follows a cubic power-curve interpolation and is capped at rated power.

PV model. Each PV string is rated at 4.4 kW. The cell-temperature correction uses NOCT = 45 degC, reference temperature 25 degC and temperature coefficient gamma = -0.0045 1/degC. Irradiance is expressed in kW/m2 with a reference value of 1.0 kW/m2.

Meteorological uncertainty. Wind uncertainty is represented by a Weibull fit and irradiance uncertainty by a Beta fit. The revised code propagates these fitted distributions into 12 stratified meteorological scenarios using reproducible seed 31415. The robust objective combines scenario mean and CVaR with weights 0.75 and 0.25 at alpha = 0.90.

4. ANN/ML renewable-generation prediction layer

Learning data. The learning target is total renewable power obtained from the physical WT/PV equations. Predictor features are sinusoidal hour and month encodings, hub-height wind speed, solar irradiance and ambient temperature. An 80/20 holdout split is used. The preferred ANN uses two hidden layers of 32 and 16 units with ReLU activations, standardization, lambda = 1e-4 and an iteration limit of 700 when fitrnet is available.

Method	RMSE (kW)	MAE (kW)	R2	MAPE (%)	Use
ANN fitrnet	1.6871	1.3043	0.99999	3.6837	Selected predictive core
LSBoost ensemble	3.1729	2.0182	0.99997	1.7556	Comparison baseline
Gaussian Process	7.6322	1.6277	0.99985	1.8450	Smooth nonlinear baseline

Selection rule. The ANN is retained because the planning loop depends primarily on absolute renewable-power accuracy (RMSE and MAE), while LSBoost has the lowest percentage error near low-generation periods.

5. ANN-assisted PSO-VoltVAR optimization

Decision vector. x = [N_WT,35, N_WT,68, N_PV,52, N_PV,70, Q52%, Q70%]. The first four variables are rounded to integers; the two Volt-VAR support variables remain continuous. In the production code the bounds are 0-12 WT units at each WT bus, 0-150 PV strings at each PV bus and 0-35% reactive support at each PV inverter.

PSO / inverter setting	Value	Meaning
Population	50	Particles per optimization run
Maximum iterations	120	Iteration budget per run
Inertia	0.90 -> 0.40	Linearly decreasing exploration weight
Velocity clamp	0.20 of variable range	Limits particle velocity
c1 / c2	1.55 / 1.55	Cognitive / social acceleration
Independent seeds	42:61	Twenty independent robust runs
Inverter loading ratio	1.10	Apparent-power capability scaling
Minimum power factor	0.90	Capability constraint
Volt-VAR breakpoints	0.94, 0.98, 1.02, 1.06 p.u.	Piecewise inverter voltage-control regions

Objective logic. Candidates are evaluated using active loss, mean voltage deviation, minimum voltage, minimum VSI, inverter capability/curtailment and capacity terms. Penalties prevent a loss-only solution from being accepted when voltage-quality or stability constraints are violated. The original IFLO values are used as benchmark anchors for the calibrated IEEE 85-bus comparative response model.

6. Verified mean-profile ablation and revised result table

Production consistency note: these are the reviewer-response mean-profile values. They are not the same object as the separate 12-scenario, twenty-run robustness results generated by the revised MATLAB code.

Case	WT	PV	VV (%)	Loss kW	VD p.u.	Vmin p.u.	Min VSI
No RES	0	0	0	118.2953	0.30662	0.92520	0.73550
Original IFLO	8	129	0	35.1939	0.06590	0.97040	0.88840
Sizing only	14	112	0	17.2465	0.05890	0.97554	0.94371
Direct equations + PSO-VoltVAR	14	112	33.08	16.3462	0.05231	0.98072	0.95850

ANN-PSO-VoltVAR	14	112	33.08	15.9888	0.05110	0.98138	0.96096
Enhanced 5-start mean-profile	12	142	33.25	15.9900	0.051082	0.98213	0.96307

Interpretation. The controlled sequence isolates the contribution of sizing, Volt-VAR support and the ANN surrogate. Sizing produces most of the active-loss decrease, while inverter support produces the clearer voltage-floor and stability-margin gains. The verified five-start point [6, 6, 71, 71, 33.25, 33.25] has essentially the same mean loss as the controlled ANN-assisted point but improves minimum voltage and VSI, which is consistent with a multi-objective rather than loss-only search.

7. Scenario-based robustness and generated numerical outputs

Twenty-run protocol. For the revised robustness analysis, the same 12 meteorological scenarios, bounds, population, iteration budget and constraint logic are used across independent seeds 42 through 61. The script exports detailed per-run decisions and metrics, summary statistics, a capacity-matched comparison, and robust component ablation. These stochastic results are intentionally kept separate from the verified mean-profile table above.

Output	Purpose
seasonal_96h_profiles.csv	Generated seasonal weather, load, RES and electrical profiles
model_metrics.csv	ANN/LSBoost/Gaussian-process prediction errors
uncertainty_fit_parameters.csv	Weibull/Beta fitted parameters
uncertainty_scenarios.csv	Scenario wind/solar factors
PSO_20run_detailed.csv / PSO_20run_statistics.csv	Independent-run evidence and summary statistics
capacity_matched_comparison.csv	Controlled comparison at equal installed WT/PV capacity
reviewer_ablation_recomputed_robust.csv	Scenario-based component ablation
bus_level_injections.csv	Bus-level active/reactive injections and inverter capability information
Supplementary_Data_Sheet_1.xlsx	Consolidated workbook exported automatically by Supplementary Code 1

8. Grid-response scope and exact validation

Calibrated IEEE 85-bus comparison. The manuscript comparison is benchmark-calibrated because the complete IEEE 85-bus branch/load data and original IFLO implementation were not available in the source material used for the extension. The calibrated response is anchored to the reported no-RES and IFLO benchmark values, so all compared cases are evaluated on the same response model. If the complete IEEE 85-bus data become available, the response function can be replaced by an exact backward/forward-sweep load-flow solver without changing the ANN-PSO-VoltVAR planning chain.

Independent exact solver check. Supplementary Code 1 additionally runs an exact IEEE 33-bus backward/forward-sweep base-case validation. The implementation asserts active loss approximately 202.6771 kW (within 0.10 kW) and minimum voltage approximately 0.91309 p.u. (within 5e-4 p.u.). This check validates the exact radial load-flow routine; it is not presented as a replacement for the calibrated IEEE 85-bus manuscript benchmark.

9. Reproduction procedure

1. Place Supplementary_Code_1_ANN_PSO_VoltVAR.m in a writable MATLAB working directory.
2. Use MATLAB R2021a or later; Statistics and Machine Learning Toolbox is recommended for fitrnet, fitrensemble and fitrgp. The script contains fallbacks where possible.
3. Run the script. If InSalah_NASA_POWER_2012_2021_hourly.csv is absent, the script requests the NASA POWER hourly file; otherwise it reuses the local copy.
4. The script builds the 96-hour profiles, fits Weibull/Beta uncertainty models, computes WT/PV power, trains the predictive models, executes robust ANN-PSO-VoltVAR optimization, generates figures and numerical CSV files, and runs IEEE 33-bus validation.

5. At the end of the run, Supplementary_Data_Sheet_1.xlsx is written automatically into the results directory together with the individual CSV outputs.

The three production files are designed to be uploaded together. Supplementary Code 1 is the executable source, Supplementary Data Sheet 1 is the tabulated numerical companion, and this Method Note explains the reproducibility scope and the distinction between deterministic mean-profile evidence and scenario-based robustness.
